# Full-Genome Sequences of Influenza A(H1N1)pdm09 Viruses Isolated from Finnish Patients from 2009 to 2013

**DOI:** 10.1128/genomeA.01004-13

**Published:** 2014-01-16

**Authors:** Triin Lakspere, Janne Tynell, Minttu Kaloinen, Maarten Vanlede, Alun Parsons, Niina Ikonen, Hannimari Kallio-Kokko, Anu Kantele, Pirkko Mattila, Henrikki Almusa, Ilkka Julkunen, Denis Kainov, Laura Kakkola

**Affiliations:** a The Institute for Molecular Medicine Finland (FIMM), Helsinki, Finland; b The National Institute for Health and Welfare, Helsinki, Finland; c Helsinki University Hospital Laboratory, HUSLAB, Helsinki, Finland; d Helsinki University Central Hospital, HUCS, Helsinki, Finland; e Department of Virology, University of Turku, Turku, Finland

## Abstract

Here we report full-length sequencing of the first large set of influenza A(H1N1)pdm09 virus genomes isolated in Finland between the years 2009 and 2013 and discuss the advantages and needs of influenza virus sequencing efforts.

## GENOME ANNOUNCEMENT

Influenza A viruses (IAV) are globally distributed pathogens causing annual epidemics and pandemics (http://www.who.int/influenza/en/). So far, six pandemics have occurred (1). The latest one was announced by the WHO in 2009 (http://www.who.int/influenza/en/). The IAV genome consists of 8 single-stranded RNA (ssRNA) segments and encodes 12 proteins: hemagglutinin (HA), M proteins (M1 and M2), neuraminidase (NA), nucleocapsid protein (NP), nonstructural proteins (NS1 and NS2), and polymerase subunits (PA, PA-X, PB1-F1, PB1-F2, and PB2). IAVs are subtyped based on sequences of surface glycoproteins HA (H1 to H17) and NA (N1 to N9) (2, 3). The recent 2009 pandemic was caused by the H1N1 subtype [A(H1N1)pdm09].

Here we report the whole-genome sequencing of 135 influenza A(H1N1)pdm09 viruses isolated from nasopharyngeal aspirates (NPAs) of Finnish patients from 2009 to 2013. The viruses from NPAs were propagated in MDCK cells. RNA was prepared for sequencing either by (i) sedimentation of the viruses from supernatants, purification with an RNeasy Plus Minikit (Qiagen), reverse transcription to cDNA with SuperScript II reverse transcriptase (Life Technologies) and random hexamers (New England BioLabs), and preparation of an RNAseq library with Illumina compatible Nextera Technology (Epicentre) (Genbank numbering starting with JQ) or by (ii) extraction of viral RNA with an RNeasy 96 kit (Qiagen) and amplification with reverse transcription (RT)-PCR as described previously (4). DNA libraries were prepared using an Illumina Nextera DNA Sample Preparation kit (GISAID and Genbank numbering starting with KF). Sequencing was done using an Illumina HiSeq2000 sequencer (100-bp paired-end reads, average 3,686,912 reads/isolate). Reads were aligned with BWA software against the reference genome A/California/07/2009. Alignment was cleaned using Picard and GATK toolkits and analyzed with Samtools to detect variants. Sequences were analyzed with BioEdit and ClustalW software.

All 135 genomes were unique compared to the reference strain A/California/07/2009. Eight viruses that originated from subsequent samplings of four patients (two samplings per patient) differed in amino acid sequences, indicating fast mutation rates. In total, 4,657 amino acid changes were identified. Viral HA, NA, and PB2 were the most frequently mutated (1,111, 671, and 753 amino acid substitutions, respectively). Several changes were identified at the antigenic sites of viral HA (T89A, N142D, A158T, K180I, D185N, G187R, S202T, A203T, N211S, S220T, and R238K; numbering with the signal peptide), which may allow viruses to escape host immunity (5, 6). Polymorphism at HA D222 (numbering without the signal peptide) was not detected (7). All virus isolates had an amantadine resistance mutation (S31N) in the viral M2 protein. Oseltamivir resistance mutations were not detected; however, isolate A/Helsinki/598/2013 accumulated the amino acid change E119K at NA during propagation. Mutations at the same position (E119V) have already been linked to oseltamivir resistance in the N2 subtype. Isolate A/Helsinki/P14/2009 encodes an NS1 protein with a truncated C terminus (203 versus 219 amino acids, confirmed by Sanger sequencing in the original sample).

Next-generation sequencing allows identification of IAV quasispecies in patient samples. However, only the most dominant variants are submitted to Genbank. We suggest that variants be included in submitted sequences. This information could be used to enhance preparedness for upcoming epidemics/pandemics.

### Nucleotide sequence accession numbers.

The whole-genome sequences of 135 A(H1N1)pdm09 isolates from Finland (years 2009 to 2013) have been deposited in Genbank (accession no. JQ409139.1 to JQ409246.1, JQ409131.1 to JQ409238.1, JQ409123.1 to JQ409230.1, JQ173145.1 to JQ173152.1, JQ173153.1 to JQ173160.1, JQ173161.1 to JQ173168.1, and KF559358 to KF560309) and GISAID (isolate identification no. EPI_ISL_145286 to EPI_ISL_145302).
